# Enhanced inflammasome activation and reduced sphingosine-1 phosphate S1P signalling in a respiratory mucoobstructive disease model

**DOI:** 10.1186/s12950-020-00248-2

**Published:** 2020-04-21

**Authors:** Hai B. Tran, Matthew G. Macowan, Adrian Abdo, Martin Donnelley, David Parsons, Sandra Hodge

**Affiliations:** 1 Department of Thoracic Medicine, Royal Adelaide Hospital, Adelaide, Australia; 2 Adelaide Medical School, University of Adelaide, Adelaide, Australia; 3 Respiratory and Sleep Medicine, Women’s and Children’s Hospital, Adelaide, Australia; 4 Robinson Research Institute, University of Adelaide, Adelaide, Australia

**Keywords:** Inflammasome, Sphingosine-1 phosphate, Respiratory muco-obstructive diseases, Cystic fibrosis, Mouse model

## Abstract

**Background:**

Inflammasomes and sphingosine-1-phosphate (S1P) signalling are increasingly subject to intensive research in human diseases. We hypothesize that in respiratory muco-obstructive diseases, mucus obstruction enhances NLRP3 inflammasome activation and dysregulated S1P signalling.

**Methods:**

Lung tissues from mice overexpressing the beta-unit of the epithelial sodium channel (βENaC) and their littermate controls were examined by histology, immunofluorescence and confocal microscopy, followed by ImageJ quantitative analysis.

**Results:**

Lower airways in βENaC mice showed patchy patterns of mucus obstruction and neutrophil-dominant infiltrations. In contrast to a ubiquitous distribution of TNFα specks, significantly (*p* < 0.05) increased specks of bronchiolar NLRP3, IL-1β, and IgG in the βENaC mouse lungs were localized to the vicinity of mucus obstruction sites. Bright Spinster homologue 2 (SPNS2) at the epithelial apex and positive correlation with sphingosine kinase 1 (SPHK1) (R^2^ = 0.640; *p* < 0.001) supported the normal bronchial epithelium as an active generator of extracellular S1P. SPNS2 in βENaC mice was sharply reduced (38%, *p* < 0.05) and lost apical localization at sites of mucus obstruction. A significant (34%; *p* < 0.01) decrease in epithelial SPHK2 was also noted at mucus obstruction sites.

**Conclusion:**

These results support that mucus obstruction may enhance NLRP3 inflammasome activation and dysregulated S1P signaling.

## Introduction

A common feature in cystic fibrosis (CF), chronic obstructive pulmonary disease (COPD), primary ciliary dyskinesia and non-CF bronchiectasis is airway obstruction resulting from overproduction and dehydration of mucus, giving these diseases the collective term ‘muco-obstructive lung diseases’ [1]. Mucus obstruction causes local hypoxia, favoring proliferation of facultative anaerobic pathogens including *Pseudomonas aeruginosa*, *Staphylococcus aureus*, and *Haemophilus influenzae* which maintain chronic airway inflammation and trigger acute flares of the diseases. The hypoxic tissue milieu itself can also initiate a sterile inflammatory cascade [1]. The excessive lung inflammation in muco-obstructive diseases involves elevated levels of pro-inflammatory cytokines, with IL-1β, TNFα, IL-6 and IL-8 often detected in bronchoalveolar lavage (BAL) or sputum of patients [2–4]. Maturation and release of IL-1β and other cytokines of the IL-1 class is regulated via activation of inflammasomes; cytosolic multiprotein complexes aggregated around a sentinel protein such as NLRP3 (NACHT, LRR and PYD domains-containing protein 3), NLRC4 (NLR family CARD domain-containing protein 4) and AIM2 (absent in melanoma 2). It is not always feasible to differentiate sterile from pathogen-induced inflammation in clinical presentations of muco-obstructive disease. Previous studies showed that signalling via the IL-1R/MyD88 axis is indispensable in the inflammatory response by the airway epithelium and neutrophils to mucus obstruction-associated hypoxia [5, 6]. Although activation of inflammasome(s) by mucus obstruction, upstream of the IL-1R/MyD88 signalling, is likely in these settings, little is known about the nature of the inflammasome(s) involved.

Sphingosine-1-phosphate (S1P) is a small bioactive lipid that regulates many cellular processes. Generation of S1P inside the cell from sphingosine is mediated by two sphingosine kinases (SPHKs), SPHK1 mostly in cytosol, and SPHK2 in intracellular organelles. S1P signalling leads to diverse or even opposing cellular effects, via intracellular targets, or membrane-bound receptors S1PR1–5 at the cell surface (‘inside-out’ S1P signalling [7]). In the latter pathway, S1P is exported via some membrane transporters, of which Spinster homologue 2 (SPNS2) has recently been shown to be functional in physiologic settings [8]. S1P signalling is part of the ‘sphingolipid rheostat’ regulatory system in which S1P and ceramides and sphingosine usually induce effects opposing to each other [7]. Increase in ceramides is a common feature and potential cause of the increased cell death, inflammation and susceptibility to infections in the airway of muco-obstructive diseases including CF, COPD and cigarette smoke-induced inflammation [9–12]. Relevant to a mechanistic relationship between inflammasome pathways and sphingolipid signalling, intracellular ceramides have been shown to be directly sensed by the NLRP3 inflammasome [13]. Less is known on the alteration of S1P signalling and its role in muco-obstructive diseases. Previous studies by us and others indicated complex dysregulation of the S1P signalling system in COPD (and in response to cigarette smoke) involving several components and various cell types [14–16]. Large gaps in this field remain, especially whether and how individual components of the S1P signalling system are dysregulated in diseases such as CF, COPD, non-CF bronchiectasis, and whether mucus obstruction directly contributes to this dysregulation. .

In this study we employed a well-characterized mouse model of muco-obstructive disease elicited by overexpression of the beta-unit of the epithelial sodium channel (βENaC) specifically in the airway [17] to explore the hypothesis that mucus obstruction can enhance both NLRP3 inflammasome activation and dysregulated S1P signalling.

## Materials and methods

### Antibodies

Primary antibodies included rabbit polyclonal to SPHK1 and SPHK2 (Bs-2652R and Bs-2653R, Bioss, Woburn, MA, USA, both 1:50), NLRP3 and IL-1β (sc-66,846 and sc-7884, Santa Cruz, Dallas, TX, USA, both 1:40), GLUT1 (Glucose Transporter 1, ab652, Abcam, Cambridge, UK, 1:100), goat polyclonal antibody to SPNS2 (sc-165,572, Santa Cruz, 1:40), TNFα (sc-1348, Santa Cruz, 1:50), myeloperoxidase (#2141, Osenses, Adelaide, SA, Australia, 1:100), and a mouse monoclonal antibody to neutrophil elastase (M0752, Dako GmbH, Jena, Germany, 1:100). Conjugated antibodies (1:150) were F (ab’)2 fragments of donkey IgG, obtained from Jackson ImmunoReseach (West Grove, PA, USA), anti-rabbit IgG (Alexa Fluor 594 or Alexa Fluor 488), anti-goat IgG (Alexa Fluor 488), and anti-mouse IgG (Alexa Fluor 647).

### Mouse model

Archived mouse lung tissues of βENaC-overexpressing mice (blocks from four animals, and pre-cut sections from another four, total *n* = 8) and their age/gender-matched wild type controls (blocks from three animals and pre-cut sections from another three, total *n* = 6) were retrieved from a previously published study approved by the Animal Ethics Committees at Women’s and Children’s Health Network, Adelaide, South Australia, and the University of Adelaide [18]. The mice originated from a mixed genetic background (C3H/HeN x C57Bl/6 N bred with male C3B6F1 wild-type). Mouse lungs were inflation fixed using 10% neutral buffered formalin (NBF), excised from the animals, fixed in NBF for a further 24 h, and processed into paraffin blocks.

### Quantitative immunofluorescence

Tissue sections were examined for protein expression and subcellular localization by immunofluorescence following a protocol adapted from our previous study of inflammasome activation in a mouse model of allergic inflammation [19]. Tissue sections cut at 5 μm thickness were mounted in tissue arrays (single section from each animal, 4 sections per array), so all animals could be examined in parallel and no batch controls of intensity were required. The signal intensity of each marker was controlled by antibody titrations in preliminary experiments. The fluorescence digital intensities in the confocal images were controlled by setting the laser power (0.1 to 5%), voltage and digital gain, so that when a uniform setting was applied to all samples (including the negative controls) in each batch, the dullest samples well exceeded the negative staining controls, while the brightest samples did not reach digital saturation. In each batch, the negative staining controls included sections incubated with only the mix of three secondary antibodies (anti-rabbit IgG AF594, anti-goat IgG AF488, anti-mouse IgG AF647). Ten to 20 independent bronchioles in each sample were photographed from an area of 0. 3-0.5 cm^2^ under a 60x objective of a FV3000 confocal system (Olympus Corporation, Shinzuku, Tokyo, Japan). The optical fields were selected in the DAPI channel to prevent bias. Monochromatic photographs were analysed using the ImageJ software (NIH, Bethesda, MA, USA). For quantitative analysis, the mean fluorescence intensity (MFI) of staining in the bronchial epithelia was measured from each photo and the background intensity of the relevant negative control was subtracted. A mean value from at least 10 images was then calculated for each animal. For analysis of strongly heterogeneous staining as for TNF-α, IL-1β and NLRP3, a lower threshold of intensity was set relatively high to gate out dull/moderate fluorescence (below the threshold) from bright specks, which were then counted using the “particle analysis” function, and normalized to number of nuclei counted in the DAPI channel.

### Histology

For exact histological localization of immunostaining patterns, following confocal imaging, sections were re-stained with Alcian Blue/PAS or H&E, then scanned with a NanoZoomer digital slide scanner (Hamamatsu Photonics, Hamamatsu, Shizuoka, Japan). Mucus plaques or plugs seen under confocal microscopy as intraluminal nonstructural masses were all confirmed in Alcian Blue/PAS scans. Levels of mucus obstruction were measured by percentage ratio between Alcian Blue/PAS-positive bronchiolar sites and the total number of bronchiolar sites in each lung section. Myeloid foci containing more than 20 nuclei were counted from whole scans, then normalized to lung area in square centimeters.

### Statistical analysis

Data was analysed using Wilcoxon signed-rank tests, Mann-Whitney tests, paired t-tests and Spearman’s correlation as appropriate, and were performed using Prism software (GraphPad Software Inc., California, USA).

## Results

### βENaC overexpression induced patchy mucus obstruction and neutrophil-dominant infiltration in mouse airways

**Fig. 1:**
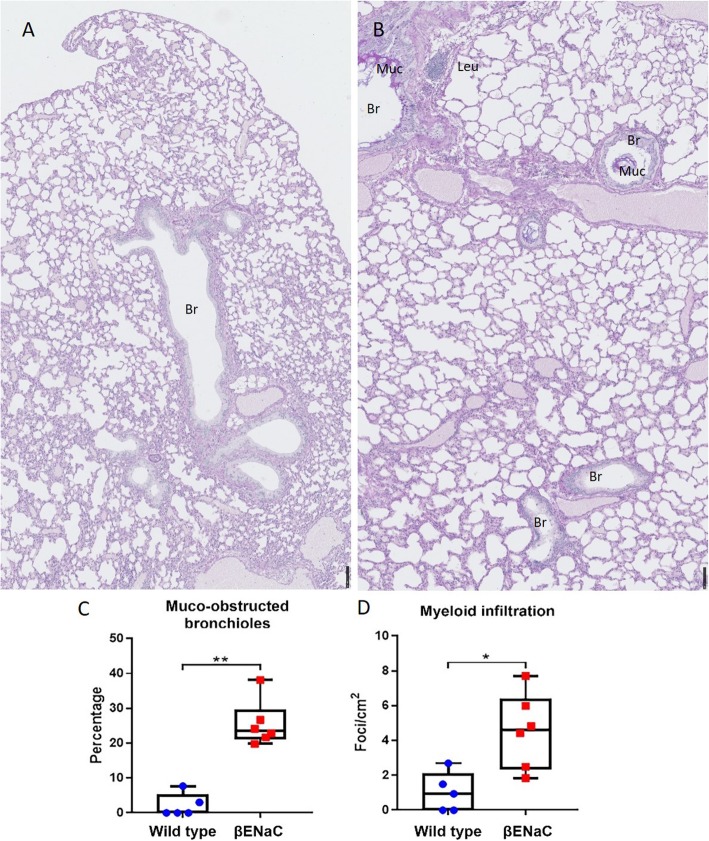
βENaC overexpression induced mucus obstruction and myeloid infiltration. **a**, **b** Representative NanoZoomer scans of Alcian Blue/PAS staining in wild type (A, *n* = 5) and βENaC (B, *n* = 6) mouse lungs. Scales are in micrometers. **c** Quantitation of mucus obstruction and (**d**) Quantitation of myeloid infiltration in wild type and βENaC mouse lungs. Dots represent mean values from individual animals. Mann-Whitney, * *p* < 0.05; ** *p* < 0.01

**Fig. 2:**
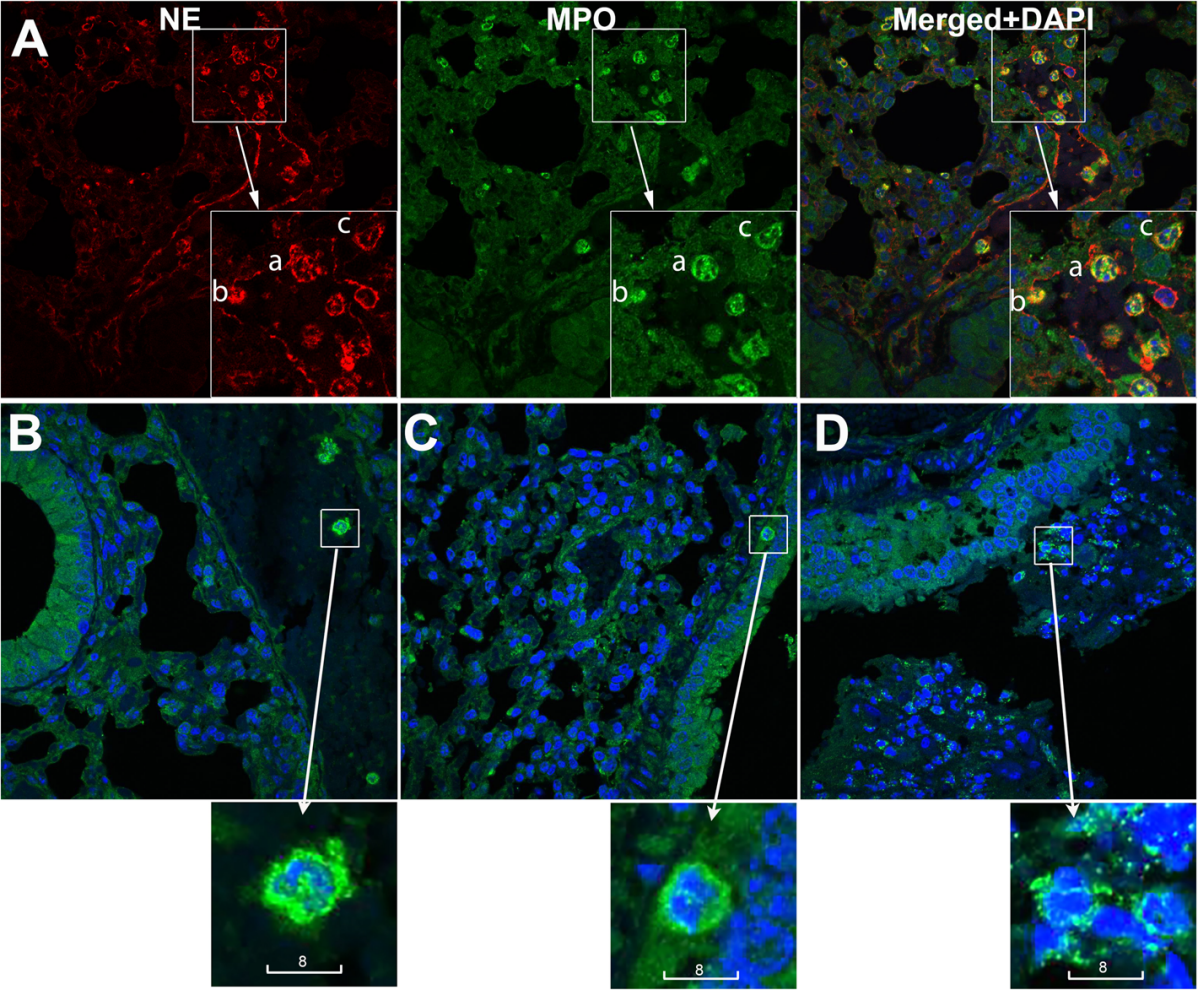
Neutrophil infiltration in the airway of βENaC-overexpressing mice. (A) Representative confocal images of neutrophils, identified by high expression of neutrophil elastase (red) and myeloperoxidase (green). Shown are neutrophils that are intravascular (a), attached to the vascular inner wall (b), and extravasated (c). (B-D) Representative confocal images of neutrophils identified by high expression of myeloperoxidase (green) and lobular shape of nuclei (Blue, DAPI). Shown are neutrophils that are intravascular in a wild type control (B), in submucosal tissue near a relatively intact bronchiole of a βENaC animal (C), and infiltrating a mucus plug in a βENaC animal (D). Scale bars are in micrometers

Comparison of βENaC-overexpressing mice to wild type controls confirmed substantial induction of airway mucus obstruction seen as plaques on bronchiolar luminal surfaces and masses plugging parts of bronchioles (Fig. 1). In accordance with our previous finding of lung function heterogeneity in βENaC mice [18], mucus obstruction in their lungs revealed patchy patterns. Within the same βENaC lung, parts of the bronchioles looked relatively intact while others were seen with mucus plaques, or entirely plugged with mucus (Fig. 1b). The βENaC lung revealed leucocyte infiltration seen as compact foci in submucosal parenchyma, and cells diffused in mucus (Fig. 1b). Quantitative analysis of whole NanoZoomer scans confirmed statistically significant increases in both mucus obstruction and infiltration foci in βENaC lungs compared to controls (Fig. 1c, d). Using neutrophil markers myeloperoxidase and neutrophil elastase, leucocyte infiltration of mucus plaques and plugs was shown to be neutrophil-dominant (Fig. 2). Levels of airway mucus obstruction and inflammatory infiltration of individual mice are given in Table S1, together with their genotypes, genders, body mass, and age at termination.

### NLRP3 inflammasome activation but not TNFα in the lungs of βENaC mice was associated with patchy mucus obstruction

**Fig. 3:**
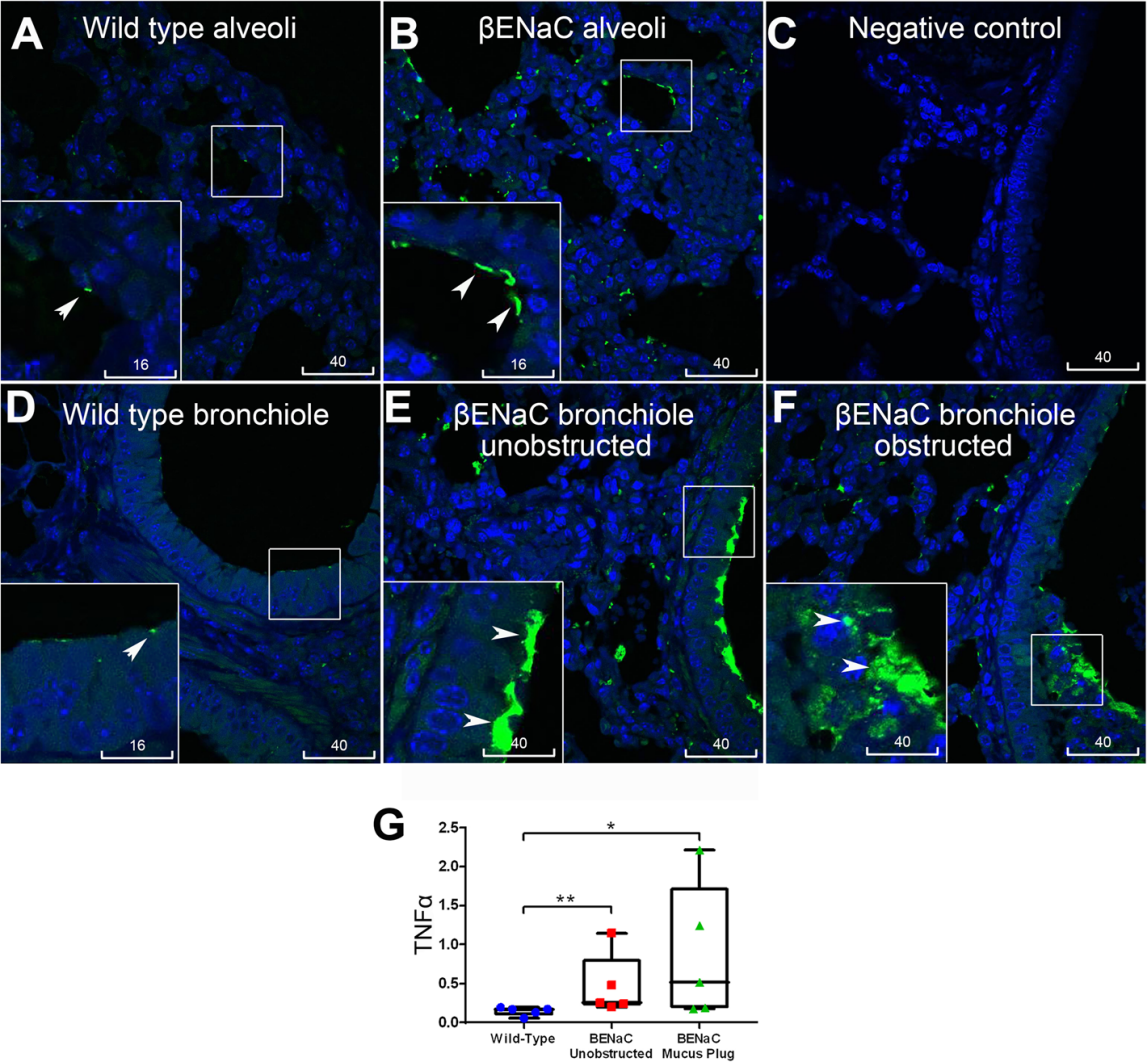
Global increase in TNF-α in the βENAC mouse lung was not associated with mucus obstruction. TNF-α was labelled in green, nuclei counterstained with DAPI (blue), scale bars are in micrometers, insets are magnification of the boxed areas. Wild type control alveoli (**a**) and bronchiolar epithelium (**d**) showed few small size specks of TNF-α (arrowheads). βENAC alveoli (**b**) and bronchiolar epithelium (**e**, **f**) showed increased TNF-α specks, irrespective of being unobstructed (**e**) or obstructed (**f**) with mucus. **c** Negative staining control. **g** Box plot represents mean values of bronchioles’ speck numbers normalized to numbers of nuclei, measured from individual mice; βENaC animals are present with two sets of data corresponding to unobstructed (red) and obstructed (green) areas. Each dot in the box plots represent the average of 10 bronchioles. Mann-Whitney, ** *p* < 0.01

**Fig. 4:**
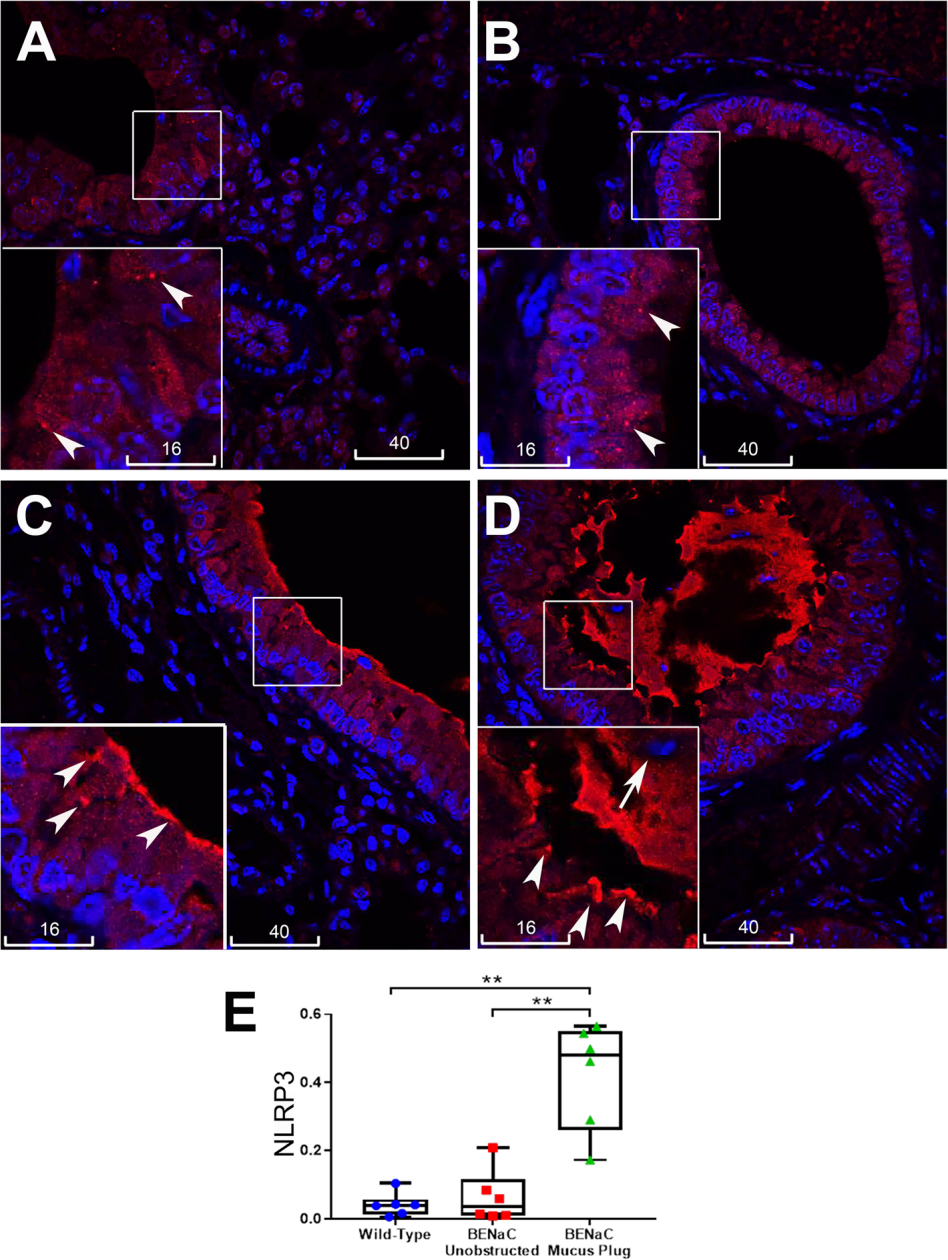
Speck immunofluorescence of NLRP3 was associated with mucus obstruction in βENaC airways. **a**-**d** Representative confocal images of NLRP3 (red). **a** Wild type control tissue showed mostly homogeneous NLRP3 staining and few sporadic small size specks of NLRP3 (arrowheads, inset is magnification of the boxed area). **b** βENaC unobstructed bronchiolar epithelium showed no significant increase in specks (arrowheads) compared to wild type control. **c** βENaC bronchiolar epithelium near a mucus plaque showed increased speck staining near and at the apical surface (arrowheads). **d** βENaC bronchiole plugged with mucus showed increased speck staining at the epithelial apex (arrowheads), and in the mucus plug which contained leucocytes (short arrow). For confirmation of mucus plaques/plugs in B-D please refer to Supplementary Data Figure S1. Nuclei counterstained with DAPI (blue), scale bars are in micrometers. **e** Box plot represents mean values of bronchioles’ speck numbers normalized to numbers of nuclei, measured from individual mice; βENaC animals are present with two sets of data corresponding to unobstructed (red) and obstructed (green) areas. Each dot in box plots represent the average of 10 bronchioles. Mann-Whitney, ** *p* < 0.01

**Fig. 5:**
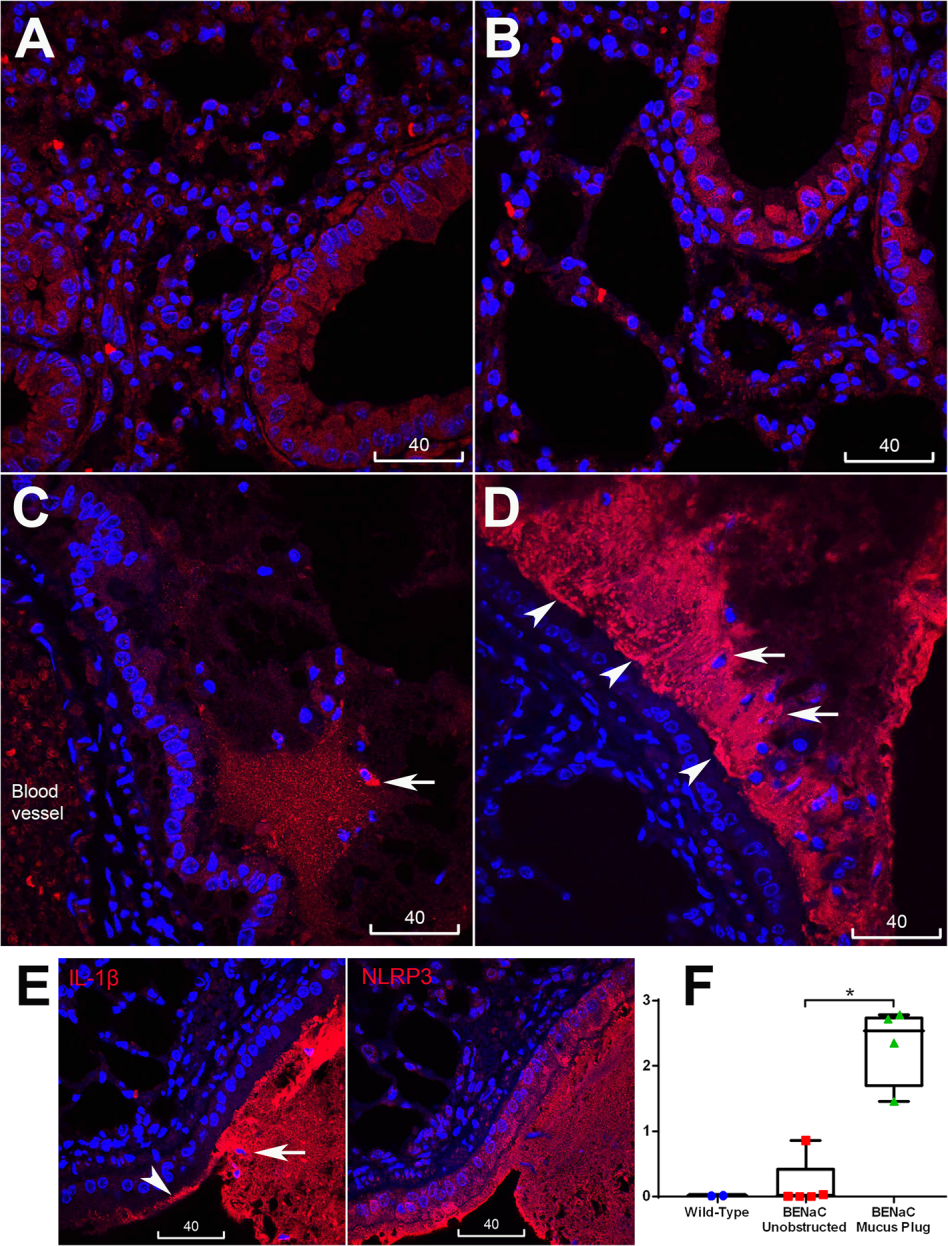
Increased immunofluorescence specks of IL-1β in bronchioles of βENaC mice associated with mucus obstruction. **a**-**d** Representative confocal images of IL-1β (red), nuclei stained with DAPI (blue), scale bars are in micrometers. Wild type control (**a**) and βENaC unobstructed bronchioles (**b**) showed mostly homogenous immunofluorescence in the epithelium. **c** and **d** βENaC obstructed bronchioles showed increased speck staining of IL-1β; (**e**) IL-1β (left) localized to the same places as NLRP3 (right) in adjacent serial sections. Short arrows indicate increased IL-1β around intraluminal leucocytes; arrowheads: increased IL-1β at epithelial surface. **f** Box plot represents mean values of bronchioles’ speck numbers normalized to numbers of nuclei, measured from individual mice; βENaC animals are present with two sets of data corresponding to unobstructed (red) and obstructed (green) areas. Each dot in box plots represent the average of 10 bronchioles. Mann-Whitney, * *p* < 0.01

We next studied pro-inflammatory changes in lungs of βENaC-overexpressing mice by quantitative immunofluorescence analysis of IL-1β and TNFα localization. As normal mouse airway epithelia always show dull/moderate staining of cytokines and inflammasome proteins, quantitative particle analysis by ImageJ was applied to screen in only bright immunofluorescence specks. In line with previous findings that lung inflammation in βENaC-overexpressing mice is driven by TNFα [20], increased bright speck staining of this cytokine was detected globally in the lungs of βENaC-overexpressing mice compared to control animals. Bright TNFα staining was localized to alveoli and bronchioles, irrespective of mucus obstruction (Fig. 3). Compared to TNFα, distribution of NLRP3 and IL-1β specks showed a marked difference. While the wild type control bronchiolar epithelia (as well as unobstructed bronchiolar epithelia of the tested βENaC animals) revealed only small, sporadic specks with predominantly homogenous fluorescence of NLRP3, increased speck staining for NLRP3 was detected at the apical epithelial surface near mucus plugs and plaques, and in intraluminal mucus masses (Fig. 4 and Fig. S1). Similar patterns of increased speck immunofluorescence associated with mucus obstruction were observed for IL-1β, which was shown localized to the same site as NLRP3 in consecutive serial sections (Fig. 5). Infiltrating cells in mucus plaques and plugs were often seen associated with increased NLRP3 and IL-1β (Figs. 4 and 5). The epithelium at and near the mucus obstruction sites displayed a trend to increased apical immunofluorescence of glucose transporter-1 (GLUT1, a marker of hypoxia), while endogenous IgG was highly distributed in mucus plaques and plugs (Figs. S2, S3). Negative control staining inserted in each batch showed no nonspecific binding of conjugated antibodies to mucus and other tissues (Fig. S4). Thus, unlike the increase in TNFα specks which showed global distribution in βENaC airways, increased specks of NLRP3, IL-1β, and intraluminal IgG were confined to airways at or near sites of mucus obstruction.

### βENaC overexpression induced mucus obstruction-associated reduction of SPNS2 expression

**Fig. 6:**
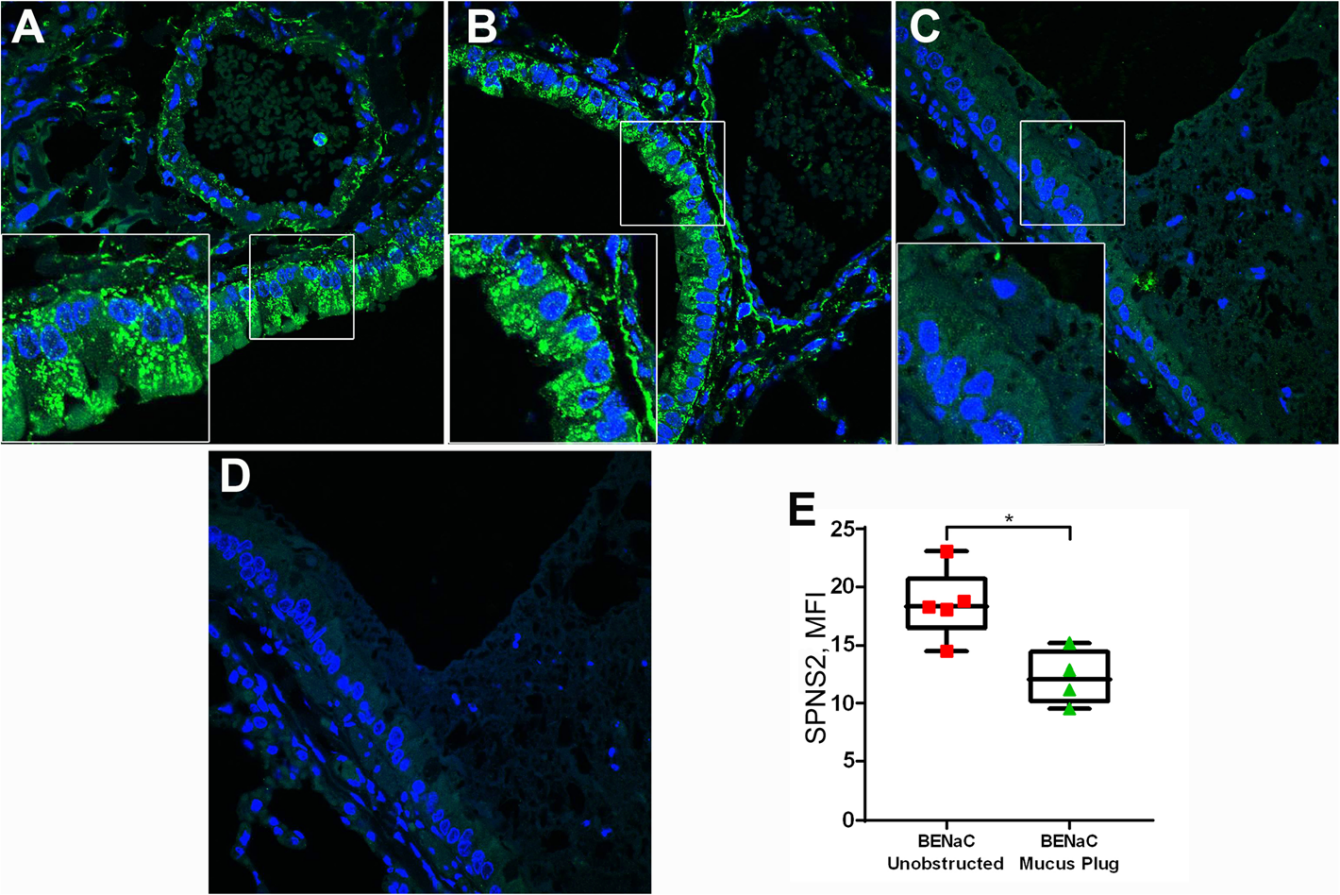
βENaC overexpression induced reduction of bronchiolar epithelial SPNS2 expression associated with mucus obstruction. **a**-**d** Representative confocal images of SPNS2 (green) in (**a**) wild type control, (**b**) βENaC unobstructed, versus (**c**) obstructed bronchiole; (**d**) a negative staining control showing no binding of AF488-conjugated anti-goat IgG. Blue is labelling of nuclei by DAPI. Scale bars are in micrometers. **e** Box plot represents MFI values of SPNS2 expression measured from individual βENaC mice, unobstructed (red) versus mucus obstructed (green) bronchioles. Mann-Whitney, * *p* < 0.05. Each dot represents the average of 10 bronchioles

The most abundant expression of SPNS2 protein in mouse lungs was localized to bronchial and bronchiolar epithelium where it was detected as dotty immunofluorescence near the lateral membranes and as intense patches at the epithelial apex. Measurement of SPNS2 immunofluorescence in bronchiolar epithelia varied greatly within each animal and among animals of the same genotype, reaching no statistically significant changes in mean intensity between the βENaC and control mice. However, on further analysis, a significant reduction in MFI was demonstrated in bronchiolar epithelia associated with mucus obstruction, compared to unobstructed epithelia (Fig. 6).

### Bronchiolar epithelial SPHK1 was co-localized and correlated with SPNS2

**Fig. 7:**
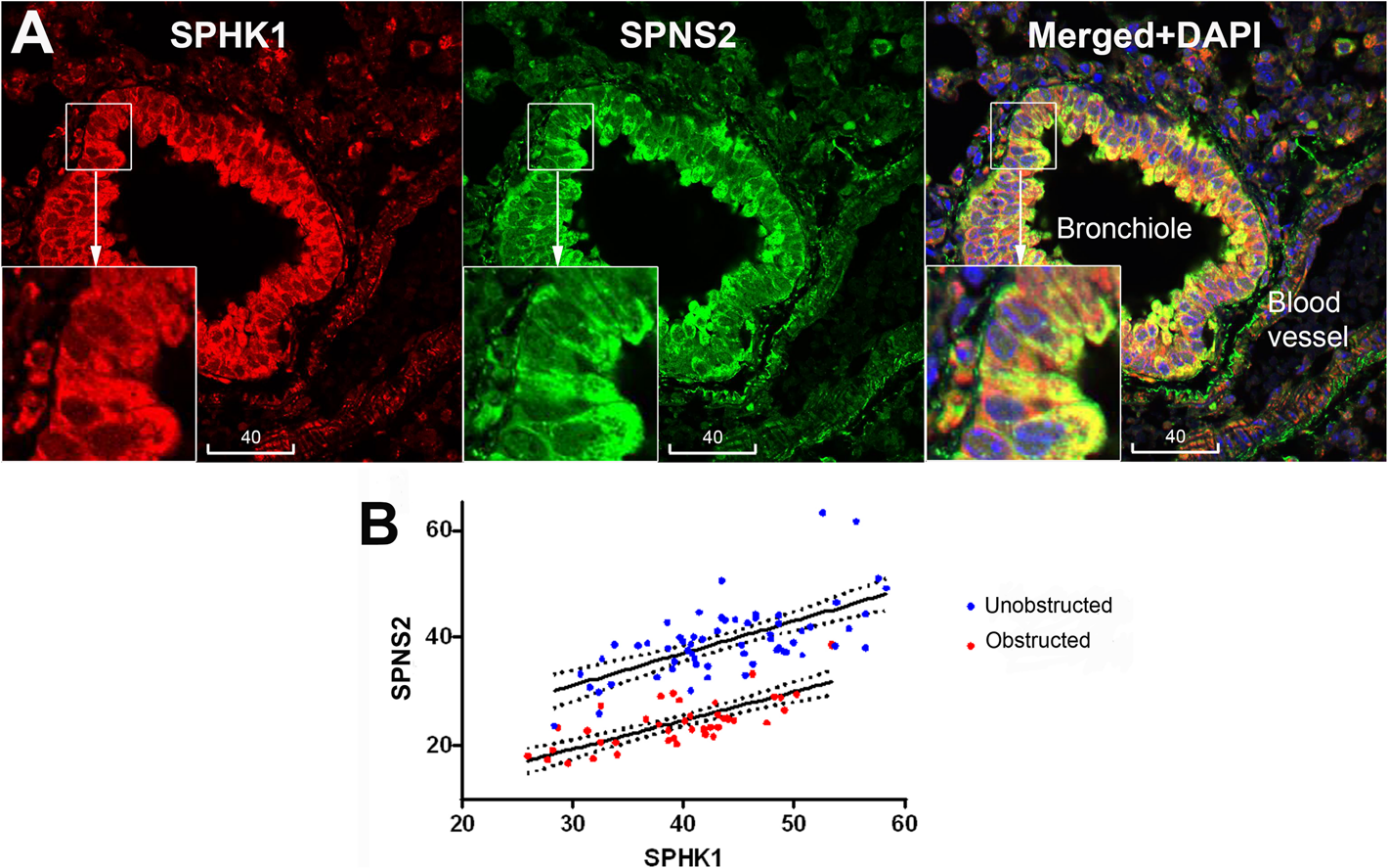
Colocalization and correlation of SPHK1 expression with SPNS2. **a** Representative confocal images of a control animal bronchiole showing colocalization of SPHK1 (red) and SPNS2 (green). Yellow is merged color of red and green. Blue is DAPI. Scale bars are in micrometers. **b** Positive Spearman’s correlation between MFI of SPHK1 and SPNS2 in both unobstructed (red; r = 0.640; *p* < 0.001; *n* = 62 bronchioles from 7 mice) and mucus obstructed (green; r = 0.745; p < 0.001; *n* = 42 bronchioles from 4 mice)

The wild type control and βENaC mouse lungs showed ubiquitous expression of SPHK1, with the brightest immunofluorescence localized to bronchiolar epithelium. In addition, moderate staining was also detected in vascular endothelium and smooth muscle, alveolar macrophages and unidentified leucocytes (Fig. 7). Of note in the bronchioles, particulate immunofluorescence of SPHK1 was co-localized with SPNS2 and was most intense at the epithelial apex (Fig. 7a). Quantitative analysis revealed a significant positive correlation between SPHK1 and SPNS2 (Fig. 7b). Epithelial expression of SPHK1 in βENaC mice showed no significant change compared to control animals, or change associated with mucus obstruction (Fig. S5).

### βENaC overexpression induced downregulation of bronchiolar epithelial SPHK2

**Fig. 8:**
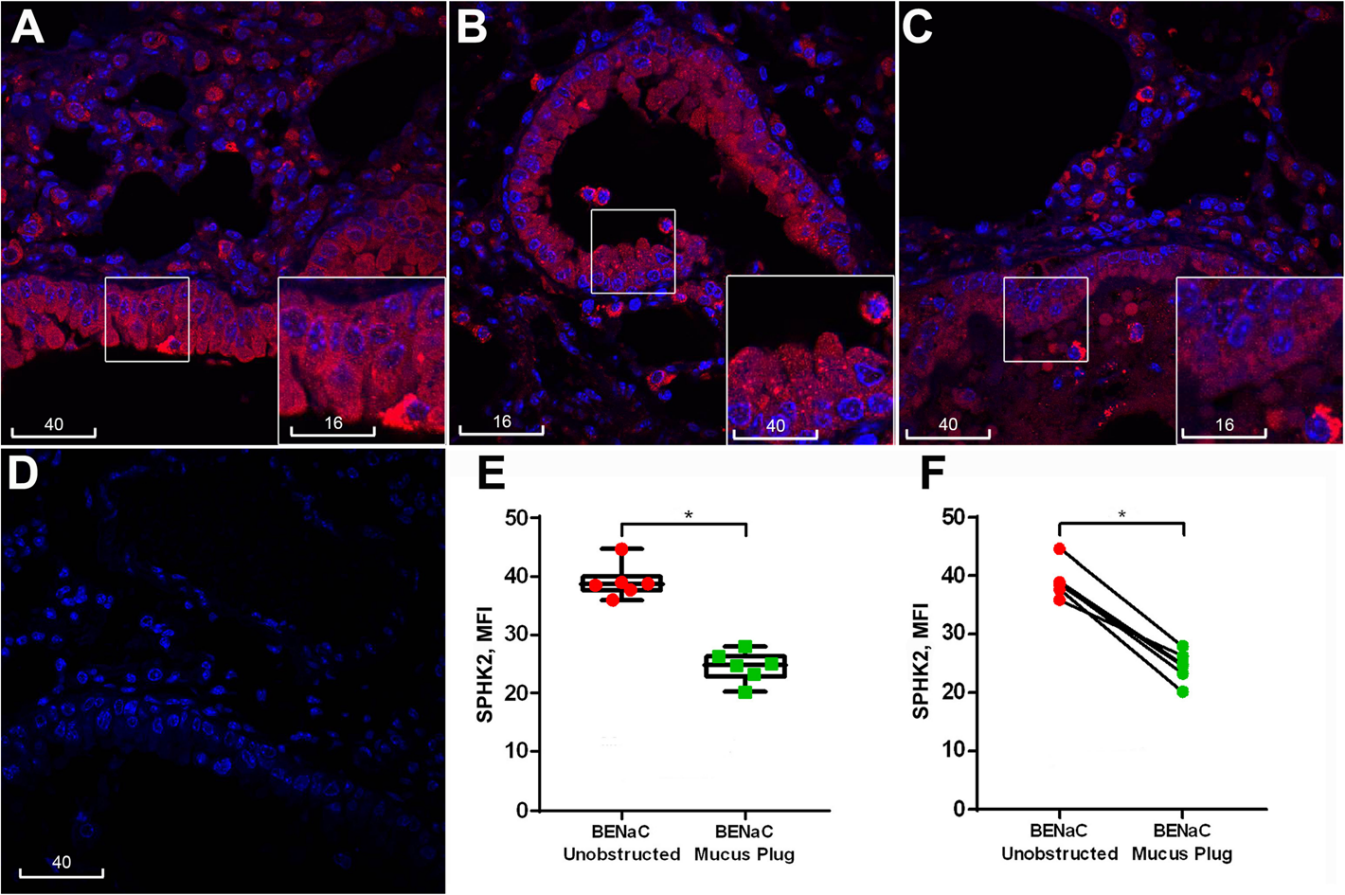
βENaC overexpression-induced reduction of bronchiolar epithelial SPHK2 expression associated with mucus obstruction. **a**-**c** Representative confocal images of SPHK2 immunofluorescence (red) in control (**a**) and βENaC mouse lungs, unobstructed (**b**) vs. obstructed airway (**c**). **d** A negative staining control shows no binding of AF594-conjugated anti-rabbit IgG (red). The insets are magnification of boxed areas. Blue is DAPI. Scale bars are in micrometres. **e** Box plot represents MFI values of SPHK2 expression measured from individual βENaC mice in unobstructed (red) versus mucus obstructed (green) epithelia (Mann-Whitney, p < 0.01). **f** Dot plot showing the same data paired for each βENaC mouse (paired t-test, p < 0.05). Each dot in E and F represent the average of 10 bronchioles

Bright immunofluorescence of SPHK2 was observed in distal airways of both control and βENaC mice, localized to alveolar walls and alveolar macrophages (Fig. S6). SPHK2-bright cells were also often detected near bronchiolar epithelial apices or in luminal mucus (Fig. 8, insets). Moderate cytoplasmic staining of SPHK2 was shown throughout the bronchiolar epithelial layers with punctate dots revealed in a subpopulation of cells (Fig. 8, inset). Quantitative analysis of bronchiolar epithelia revealed a non-significant trend of reduction of SPHK2 in βENaC mice compared to controls, and a significant reduction in mucus obstruction-associated epithelia compared to those without mucus obstruction (Fig. 8).

## Discussion

**Fig. 9:**
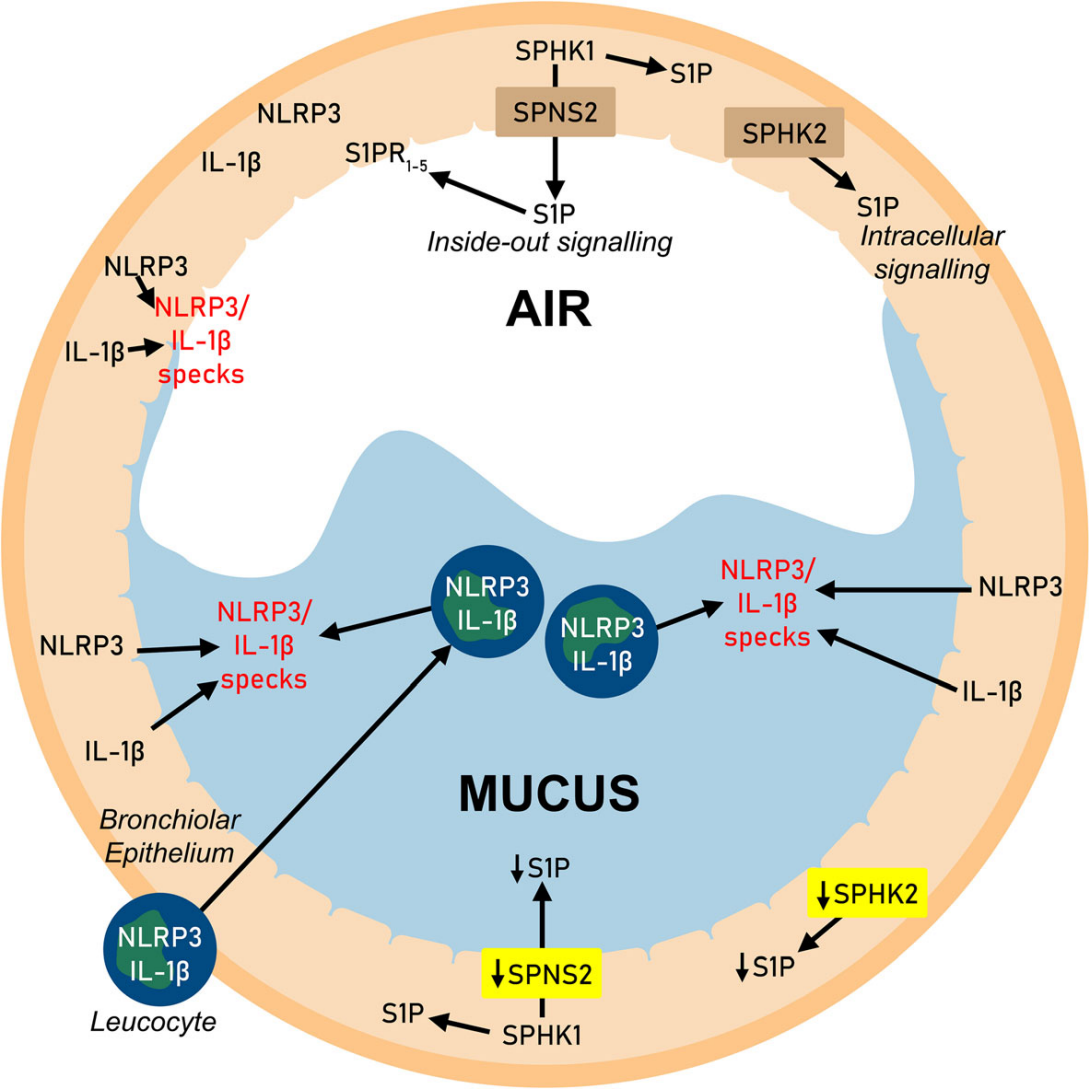
Hypothetical model of NLRP3 inflammasome activation and S1P signalling dysregulation in mucus-obstructed bronchioles. Unobstructed bronchioles (AIR): NLRP3 is mostly in monomeric state, only background levels of cleaved IL-1β are detected; SPHK1 generates cytosolic S1P, part of which is exported via SPNS2 for extracellular (inside-out) signalling via S1PRs; SPHK2 generates S1P mostly for intracellular signalling. Obstructed bronchioles (MUCUS): Increased oligomerization of NLRP3 (inflammasome activation) in either epithelium or infiltrating leucocytes results in IL-1β cleavage and increased luminal specks of NLRP3/IL-1β; decrease of SPNS2 and SPHK2 results in reduction of extra- and intracellular S1P signalling

This study presents findings from a mouse model of airway mucus dehydration and obstruction elicited by overexpression of an epithelial ion channel, βENaC, which support a hypothesis that NLRP3 inflammasome activation and dysregulated S1P signalling are associated with mucus obstruction, the common pathologic component in CF, COPD and other muco-obstructive diseases (summarized in Fig. 9). Increased bright specks of NLRP3 and IL-1β detected in the lung of βENaC overexpressing mice were highly localized to the vicinity of mucus plaques and plugs. Specks vs. homogenous fluorescence reflects oligomeric vs. monomeric states and is a method for the detection of NLRP3 inflammasome activation [21]. In the lungs, measurement of IL-1β/IL-18 in BAL is a commonly accepted technique to detect inflammasome activation, particularly in mouse studies [22]. Due to unavailability of BAL samples, the mentioned approach could not be done in this study, although detection of IL-1β in luminal spaces or at epithelial surfaces indicated the cytokine release. Next, although apical membrane patterns of NLRP3/ IL-1β specks near obstruction sites indicate their epithelial origin, specks were seen embedded in mucus containing infiltrating neutrophils and other leucocytes, supporting a myeloid origin. A precise contribution from each cell type and/or reduced mucociliary clearance into the bright intraluminal staining of obstructed bronchioles could not be assessed from this study. The localization of NLRP3/IL-1β specks to and near mucus obstruction sites irrespectively of their origin nevertheless supported the hypothesis that NLRP3 inflammasome activation could be enhanced downstream of mucus obstruction.

IL-1α released from hypoxia-induced necrosis in non-myeloid cells has been implicated in activation of the IL-1R/MyD88 axis and initiation of neutrophilic inflammation [5, 6]. Furthermore, evidence of the NLRP3/IL-1β pathway activation in a direct association with HIF-1α was obtained from a rat model of hypoxia and thrombosis [23], prompting us to explore an association between NLRP3/IL-1β specks and local hypoxia. In this study, GLUT1 was employed as an in situ marker of hypoxia, which revealed however only a non-significant trend towards increased apical immunofluorescence near the mucus plugs. Further studies are thus required to demonstrate mucus obstruction-associated hypoxia as a causal factor in airway NLRP3 inflammasome activation. Given that immune complexes are capable of priming the NLRP3 inflammasome [24], an additional factor in NLRP3 inflammasome activation in the βENaC overexpression model could be the high concentration of IgG observed in mucus plaques and plugs.

Consistent with our findings in the lungs of mice chronically exposed to cigarette smoke [15], the mouse model of βENaC overexpression revealed bronchiolar epithelial SPNS2 downregulation, with the notable difference that SPNS2 downregulation in βENaC-overexpressing mice was localized to mucus obstruction sites. The intense and colocalized expression of SPNS2 and SPHK1 at the apex of intact bronchiolar epithelia supports this cell type as a major generator of exogenous S1P in the airways. Epithelial SPNS2 downregulation at mucus obstruction sites would lead to reduced S1P export and subsequent signalling via autocrine/paracrine ligation of S1PRs on the surface of epithelial and/or other cell types. A further important finding from this study was a significant downregulation of bronchiolar epithelial SPHK2 expression at mucus obstruction sites in the lung of βENaC over-expressing mice. In contrast to SPHK1 localization to the cytosol and near the cell membrane, SPHK2 was localized to mitochondria and nuclei, suggesting its role in regulation of intracellular autocrine production of S1P. Taken together, these findings suggest that both the ‘inside-out’ and the intracellular S1P signalling pathways are dysregulated in the lungs of βENaC mice, and that these defects are associated with mucus obstruction. Depending on the subcellular compartmentalization of S1P generation and the cell types involved, S1P signalling leads to diverse effects, including those offsetting the effects of ceramides [7, 25]. Given that ceramides are increased in muco-obstructive diseases [9–12] and that they can directly activate the NLRP3 inflammasome [13], a decrease in S1P signaling may have a role in amplification of the pro-inflammatory response.

## Conclusions

In conclusion, our results support NLRP3 inflammasome activation and S1P signalling dysregulation in the lung disease elicited by βENaC overexpression, and that both features are associated with mucus obstruction. Our data suggest that the NLRP3 inflammasome and the S1P signalling system could represent potential contributors to pathology and therefore putative therapeutic targets in CF, COPD and other muco-obstructive respiratory diseases.

## Supplementary information

**Additional file 1: Figure S1**. Localization of NLRP3 specks to mucus obstruction sites in Nanozoom scans of Alcian-Blue re-staining.

**Additional file 2: Figure S2**. GLUT1 immunofluorescence.

**Additional file 3: Figure S3**. Increased luminal staining of IgG in mucus-obstructed bronchioles.

**Additional file 4: Figure S4**. Confocal images of a representative negative staining control.

**Additional file 5: Figure S5**. Representative confocal images of SPHK1 immunofluorescence in control and mucus-obstructed airway.

**Additional file 6: Figure S6**. Representative confocal images of SPHK2 immunofluorescence in alveolar macrophages.

**Additional file 7: Table S1**. Parameters of mice with lung samples available for the study.

## Data Availability

All data generated and/or analysed during this study are included in this published article.
