# Supplementary figures and images for: Enhanced inflammasome activation and reduced sphingosine-1 phosphate S1P signalling in a respiratory mucoobstructive disease model
Source: J Inflamm (Lond). 2020 Apr 21;17:16. doi: 10.1186/s12950-020-00248-2 (PMC7175514; doi:10.1186/s12950-020-00248-2)

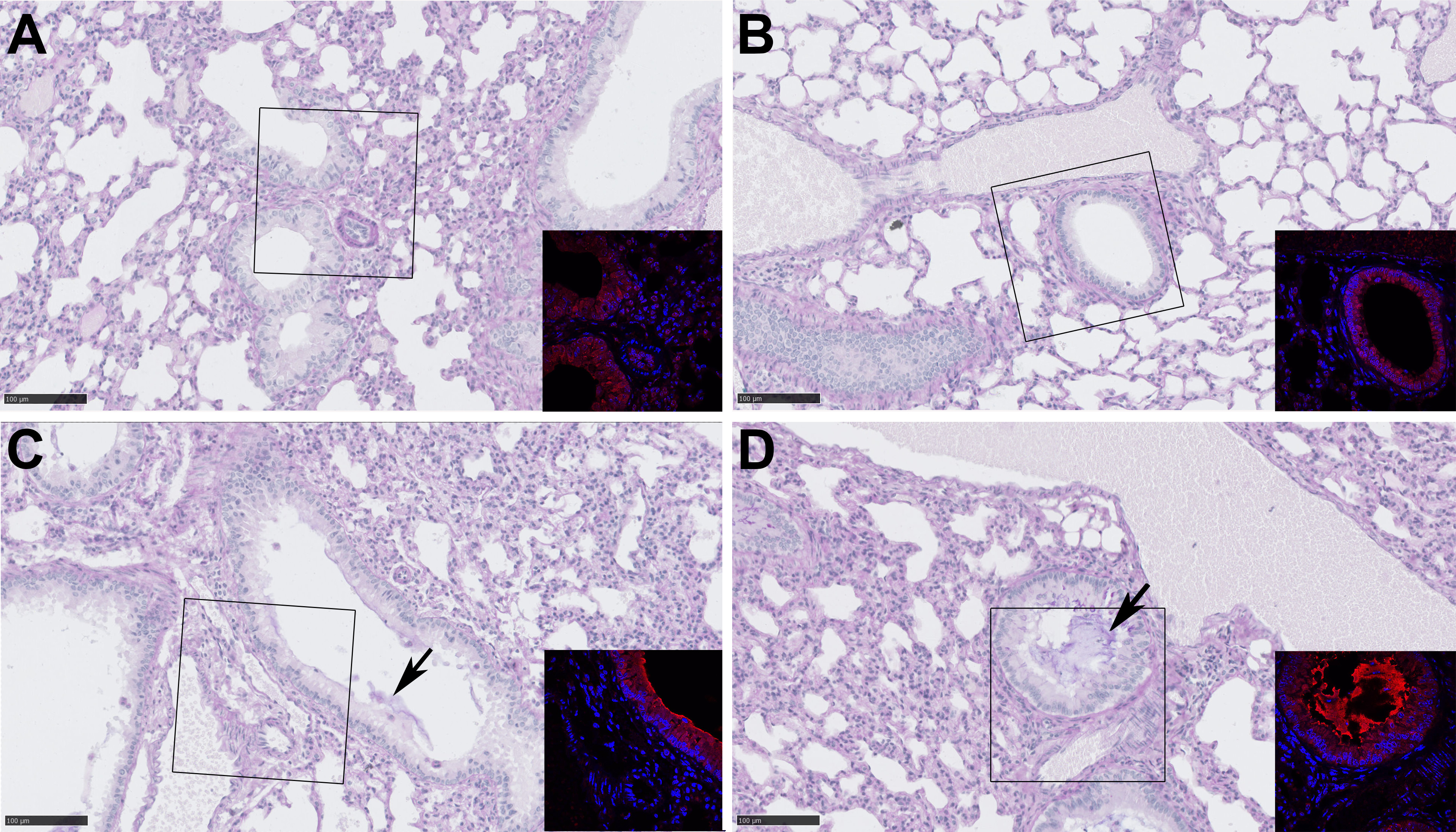

Supplement: Supplementary file 1 — Additional file 1: Figure S1. Localization of NLRP3 specks to mucus obstruction sites in Nanozoom scans of Alcian-Blue re-staining. [file 12950_2020_248_MOESM1_ESM.jpg]

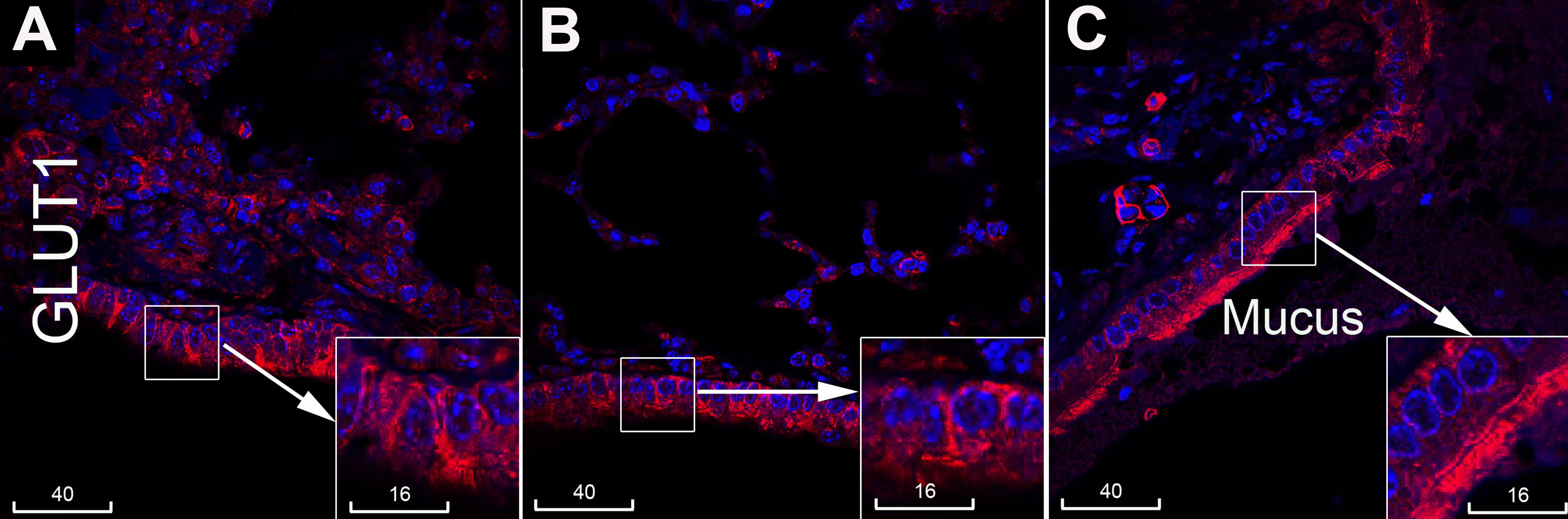

Supplement: Supplementary file 2 — Additional file 2: Figure S2. GLUT1 immunofluorescence. [file 12950_2020_248_MOESM2_ESM.jpg]

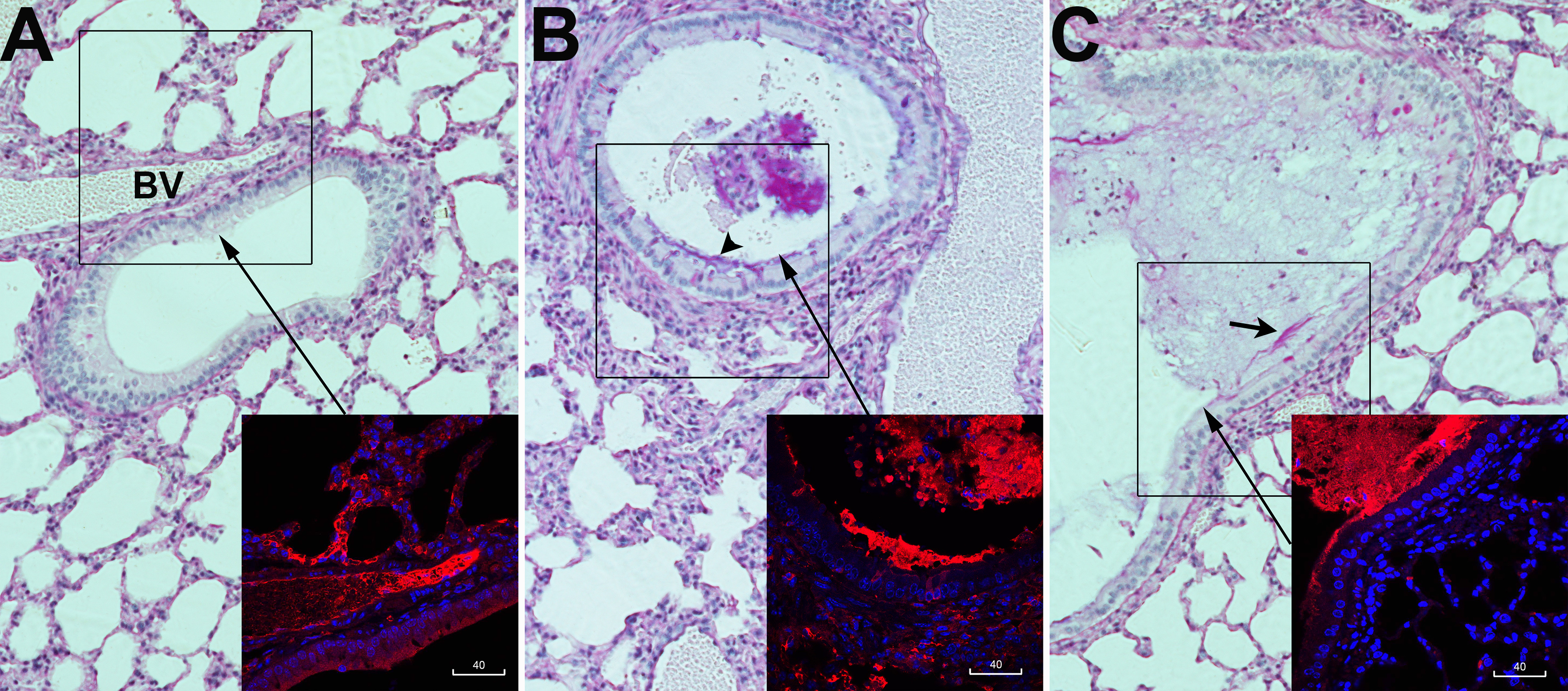

Supplement: Supplementary file 3 — Additional file 3: Figure S3. Increased luminal staining of IgG in mucus-obstructed bronchioles. [file 12950_2020_248_MOESM3_ESM.jpg]

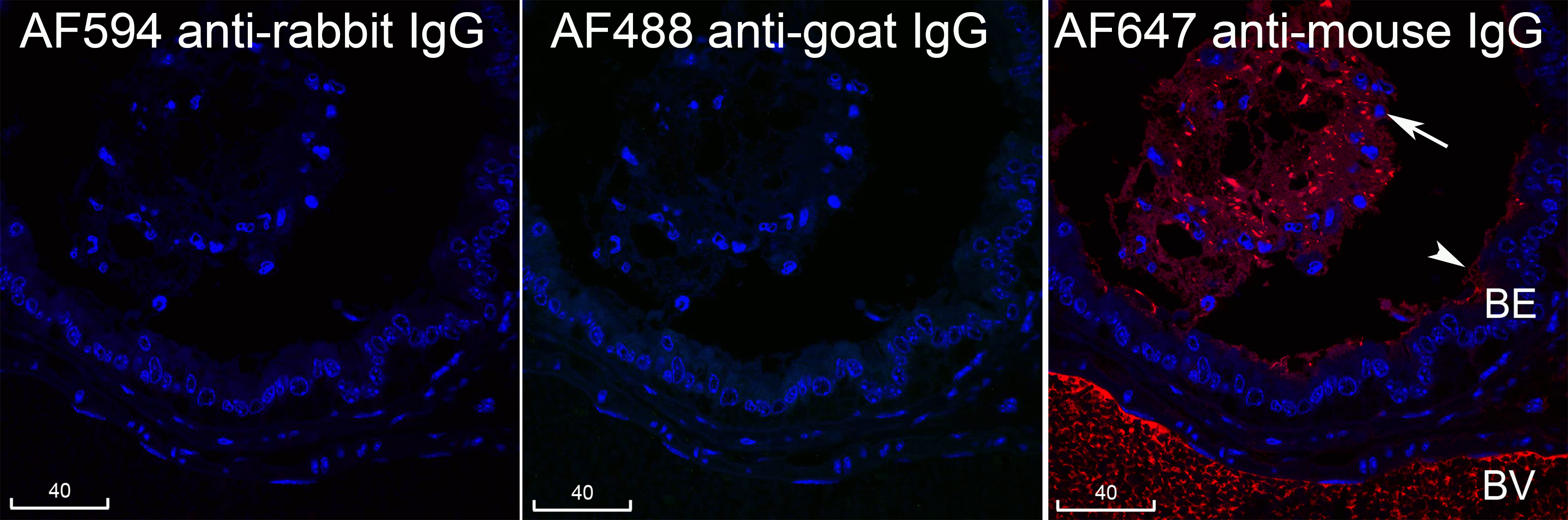

Supplement: Supplementary file 4 — Additional file 4: Figure S4. Confocal images of a representative negative staining control. [file 12950_2020_248_MOESM4_ESM.jpg]

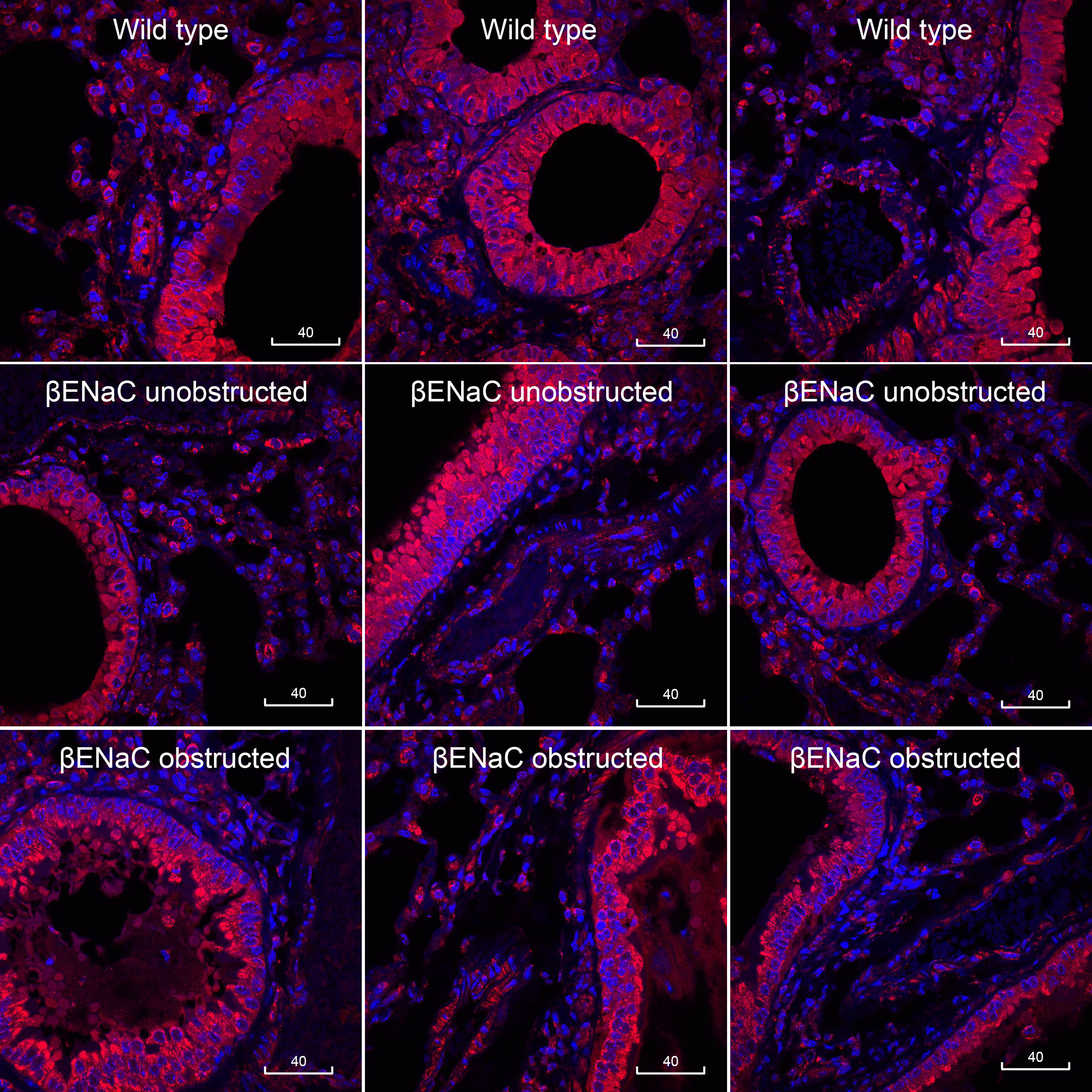

Supplement: Supplementary file 5 — Additional file 5: Figure S5. Representative confocal images of SPHK1 immunofluorescence in control and mucus-obstructed airway. [file 12950_2020_248_MOESM5_ESM.jpg]

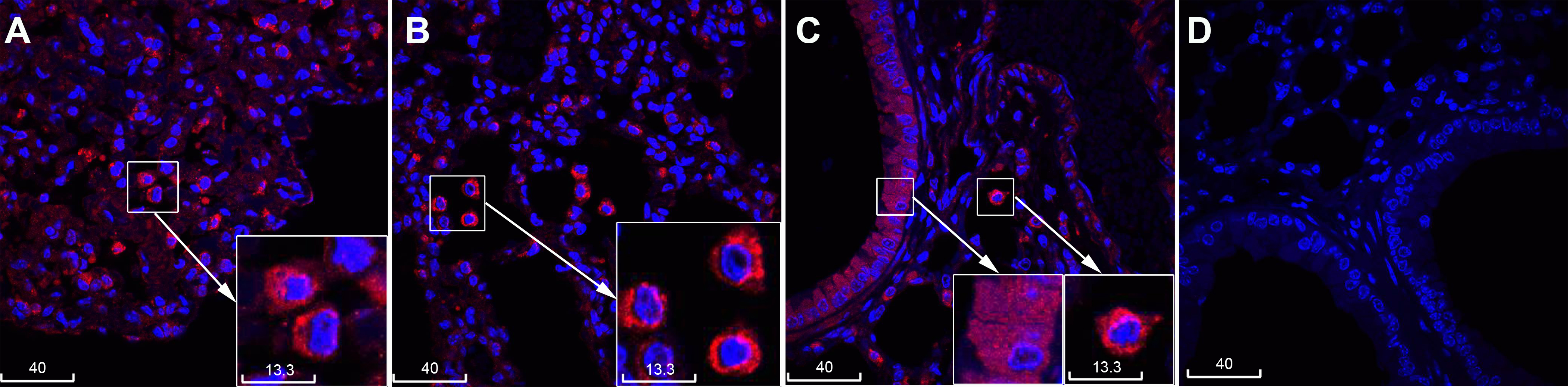

Supplement: Supplementary file 6 — Additional file 6: Figure S6. Representative confocal images of SPHK2 immunofluorescence in alveolar macrophages. [file 12950_2020_248_MOESM6_ESM.jpg]
