# Supplementary material for: Enhanced inflammasome activation and reduced sphingosine-1 phosphate S1P signalling in a respiratory mucoobstructive disease model
Source: J Inflamm (Lond). 2020 Apr 21;17:16. doi: 10.1186/s12950-020-00248-2 (PMC7175514; doi:10.1186/s12950-020-00248-2)
Supplement: Supplementary file 7 — Additional file 7: Table S1. Parameters of mice with lung samples available for the study. [file 12950_2020_248_MOESM7_ESM.docx]

**S1 Table**. **Parameters of mice with lung samples available for the study**

| ID | Genotype | Gender | Body mass (g) at termination | Age (days) at termination | Mucus obstruction, % of airways | Leucocyte infiltration, foci/cm2 |
| --- | --- | --- | --- | --- | --- | --- |
| 2 | Wild Type | F | 19.8 | 50 | 3.04 | 0 |
| 3 | βENaC | F | 17.9 | 50 | 26.70 | 4.83 |
| 5 | Wild Type | F | 18.9 | 52 | 7.68 | 0 |
| 6 | βENaC | F | 23.3 | 58 | 38.13 | 7.71 |
| 7 | Wild Type | M | 26.5 | 58 | 0 | 1.50 |
| 8 | Wild Type | M | 20.4 | 52 | 0 | 2.69 |
| 9 | Wild Type | F | 21.9 | 52 | 0 | 0.95 |
| 11 | βENaC | M | 21.6 | 50 | N.D. | N.D. |
| 14 | βENaC | M | 20.7 | 50 | N.D. | N.D. |
| 21 | βENaC | F | 18.3 | 52 | 22.88 | 4.42 |
| 27 | βENaC | M | 22.9 | 51 | 21.67 | 2.49 |
| 29 | βENaC | M | 21.3 | 52 | 24.14 | 5.99 |
| 30 | βENaC | M | 21.3 | 50 | 19.80 | 1.85 |

Mucus obstruction was evaluated in Alcian Blue/PAS staining, values displayed as number of bronchioles seen with mucus plugs per total number of bronchioles in the analysed sample. Leucocyte infiltration foci were counted as well-defined aggregations of at least 20 cells. N.D., not done.
